# Fabrication and Characterization of Electrospun Semiconductor Nanoparticle—Polyelectrolyte Ultra-Fine Fiber Composites for Sensing Applications

**DOI:** 10.3390/s111110372

**Published:** 2011-10-31

**Authors:** Jennifer S. Atchison, Caroline L. Schauer

**Affiliations:** Department of Materials Science and Engineering, Drexel University, Philadelphia, PA 19104, USA

**Keywords:** composite-nanofibers, polyelectrolytes, electrospinning, nano-effects

## Abstract

Fluorescent composite fibrous assembles of nanoparticle-polyelectrolyte fibers are useful multifunctional materials, utilized in filtration, sensing and tissue engineering applications, with the added benefits of improved mechanical, electrical or structural characteristics over the individual components. Composite fibrous mats were prepared by electrospinning aqueous solutions of 6 wt% poly(acrylic acid) (PAA) loaded with 0.15 and 0.20% v/v, carboxyl functionalized CdSe/ZnS nanoparticles (SNPs). The resulting fluorescent composite fibrous mats exhibits recoverable quenching when exposed to high humidity. The sensor response is sensitive to water concentration and is attributed to the change in the local charges around the SNPs due to deprotonation of the carboxylic acids on the SNPs and the surrounding polymer matrix.

## 1. Introduction

The development of novel approaches towards exceptionally sensitive detection techniques continues to challenge the field of chemical sensors. Currently the sensitivity of devices, which rely on molecules interacting with a surface as the detection route, is believed to benefit from an increase in surface area per unit mass. Therefore, there has been a substantial effort into making sensors that are both small and have a large surface to volume ratio [1–5]. Additionally, when one dimension of the sensor is reduced to less than 100 nm, distance dependent interactions can become a complementary sensing pathway [3,6,7]. The elegant solution to this challenge will most certainly capitalize on the selectivity and sensitivity of single or few molecule-to-surface interactions afforded by reactive organic chemistries, detection strategies such as color change or fluorescence coupled with simple, affordable but flexible fabrication techniques. Ultimately a nanoscale composite chemical sensor that is easy to fabricate, has excellent sensitivity, response time, and selectivity has applications in medical diagnostics, environmental monitoring, biological warfare, food quality assurance and industry process control [8–13].

**Figure 1.**
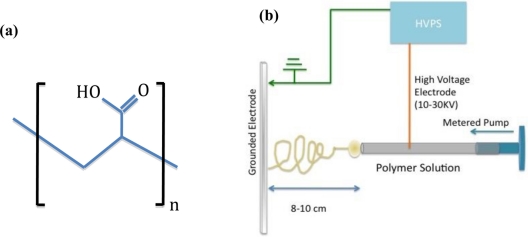
**(a)** Chemical structure of poly acrylic acid; **(b)** Schematic diagram of the electrospinning apparatus.

One nanofabrication technique that meets the above requirements is electrospinning. The electrospinning process results in non-woven mats of ultrafine fibers with diameters in the range of 20 nm to 1,000 nm that are several centimeters long, with surface areas that are 1 to 2 orders of magnitude greater than those of continuous thin films. The driving force for fiber production is a high voltage potential difference between a spinneret and a grounded target, as illustrated in Figure 1(b). As the spinning solution is pumped through the spinneret a droplet forms and is electrified by the applied high voltage. The electrified droplet deforms as the static charges repel one another creating a Taylor cone [14]. As the voltage increases the Taylor cone continues to elongate towards the grounded plate until a tendril is formed. The tendril experiences several competing forces including an opposing force due to the surface tension of the spinning solution. As long as the force due to the applied voltage exceeds the surface tension, fibers will continue to be drawn from the droplet. During the flight time to the target, the charges in the fibers are also rearranging and this leads to a bending instability [15]. As a consequence of the bending instability, the fiber undergoes a whipping motion that further reduces the diameter of the fiber. Essentially the fiber diameter and morphology is determined by the viscosity, surface tension and conductivity of the spinning solution as well as the applied electrostatic field, the distance to the target and the solution replenishment rate. In addition to the easily controllable processing parameters, electrospinning has been used to make fibers from synthetic polymers, natural polymers, polyelectrolytes and composites [16–20]. Moreover, it is expected that the large surface area, realized in these non-woven mats, have the potential to provide extraordinary sensitivity and fast response time in sensing applications.

Polyelectrolytes are a class of polymers that carry a charge on the pendant groups along the backbone chain. For most polyelectrolytes the charged group is an amine or carboxyl group, which makes them an excellent platform for pH, humidity, and metal ions sensors [17,21]. For this study, poly(acrylic acid) (PAA), [Figure 1(a)], was used as the primary sensor platform for the creation of a humidity sensor because of its ability to electrospin nanoscale fibers and its known sensitivity to humidity. PAA has been previously spun into nanofibers from an aqueous solution [22,23]. This work provided a strong starting point to investigate the properties of polyelectrolyte composite nanofibers that combine the functionality and sensitivity of the polymer matrix with the unique optical and chemical properties of semiconductor nanoparticles (SNPs).

In particular SNPs have narrow tunable emission over the visible spectrum owing to quantum confinement effects, continuous absorption profiles leading to large a Stoke’s shift, robust signal intensity, excellent photochemical stability and tailored surface chemistries [24]. They are exceptionally suited for sensing applications due to their high surface to volume ratio, surface chemistry dependent photoluminescence (PL) and sensitivity to cations and anions [8,13,25]. Furthermore SNP’s in ultra fine fibers exhibit waveguiding behavior and changes in the PL and absorption spectra suggesting additional confinement effects due to the submicron fiber diameters [26,27]. Several groups have reported optical sensors based on PL enhancement or quenching of the SNPs in both thin film geometries and ultrafine fibers but the polymer matrices for these sensors were charge neutral [28,29].

In this work we demonstrate a composite fluorescent reversible humidity sensor that combines an anionic polyelectrolyte with water-soluble carboxyl terminated SNP. These composite fibers were electrospun into non-woven mats that were exposed to different relative humidity environments and pHs.

## 2. Materials and Methods

### 2.1. Materials

Powdered poly (acrylic acid) (PAA) polymer [M_w_ ≈ 250,000 g/mol; the chemical structure is shown in Figure 1(a)] was used as provided by Sigma Aldrich. Water soluble Qdot® 525 ITK™ carboxyl terminated CdSe/ZnS SNPs (λ_em_ = 525) purchased from Invitrogen were used as received. Anhydrous toluene, triethylamine and pyridine were purchased from Sigma Aldrich and used as received. All water used in preparing samples was twice deionized to 18.2 MΩ/cm.

### 2.2. Polymer—SNP Solution Preparation

The SNP-polymer solutions were prepared by adding 15 or 20 μL of 8 μM stock solution of Qdot 525 to 10 mL of deionized water. Each suspension was sonicated for 3 min, then 2 mL of the suspensions were pipetted out and kept in reserve for spectroscopy. To the remaining 8 mL of solution, 511 mg of PAA were added for a polymer content of 6 wt%. The PAA-Qdot 525 solutions were mixed for 24 h until the polymer was completely solvated.

### 2.3. Polymer—SNP Solution Characterization

Absorption and photoluminescence (PL) spectra were taken with Ocean Optics USB 2000 UV-VIS spectrometer with a DH 2000 Deuterium Tungsten Halogen light source (absorption spectra) and UV long wavelength lamp (λ = 360 nm) for PL spectroscopy.

### 2.4. Electrospinning Apparatus

The electrospinning station consisted of a Gamma High Voltage Research power supply (0–60 KV), Harvard Apparatus 11 plus metered pump, copper screen grounded electrode, and 10 mL BD syringe with a 21 gauge needle. A diagram of the electrospinning station is shown in Figure 1(b). Fibrous mats of composite and pristine fibers were collected on aluminum foil or waxed paper.

### 2.5. Electron Microscopy

A Ziess VP 5 Supra scanning electron microscope (SEM) was used to image the fibrous mats. The SEM samples were prepared by sputter coating, Denton Vacuum, with Pt target at 40 mA for 35 s resulting in a 7–8 nm conductive film. The SEM was run at 3.5 KV at a 11 mm working distance in high vacuum. Swelling studies were completed in an FEI Environmental Scanning Electron Microscopy (ESEM) at 10 KV at a working distance of 10 mm in variable pressure between 1 and 5 Torr. Transmission electron microscopy (TEM) was performed with a Jeol JEM 2100 at 120 KV.

### 2.6. Fluorescence Microscopy

Standard wide-field fluorescence microscopy was performed using a Ziess Axioskop 2 plus with an Axi Cam camera. FITC filter set included an excitation filter centered at 470 ± 20 nm and emission filter centered 525 ± 25 nm. Images were taken with 20× (0.75 N.A.) Fluor objective.

### 2.7. Steady State and Time Resolved Fluorometry

All steady state and time resolved fluorometry measurements were performed on a PTI spectrofluorometer system. The steady state fluorometer utilized monochromatized white light excitation and emission filtering was accomplished with a monochromator. Environmental fluorometry on the non-woven composite mats was done with a Teflon custom film holder. The time resolved fluorometer delivers a nitrogen laser generated 1 ns pulse at 337 nm at a pulse repetition rate of 10 Hz. The resulting fluorescence decay curves were fitted to an exponential decay, shown below, and the excited lifetime of the SNPs were extracted from the fitted data:

(1) $$D(t)=\sum_{t}a_{i}\text{exp}\left(\frac{-t}{\tau_{i}}\right)$$

where *D*(*t*) is the delta function generated decay at time, *t*, and *τ*, is the excited lifetime of the SNP.

### 2.8. Fourier Transform Infrared Spectroscopy

A Thermo Nicolet Nexus 870 FT-IR Spectrometer with a single bounce Attenuated Total Reflection (ATR) accessory was used to take infrared spectra of the mats. The resolution was set to 4 cm^−1^ and the scans were integrated 64 times. To take the measurements the fibrous mats were removed from the substrate and secured over the ATR crystal.

### 2.9. Humidity Testing

The ambient humidity and air introduced to the samples were monitored with a portable hygrometer/thermometer (TFA Brand).

## 3. Results and Discussion

### 3.1. Electrospinning SNP-PAA Composite Fibers

**Figure 2.**
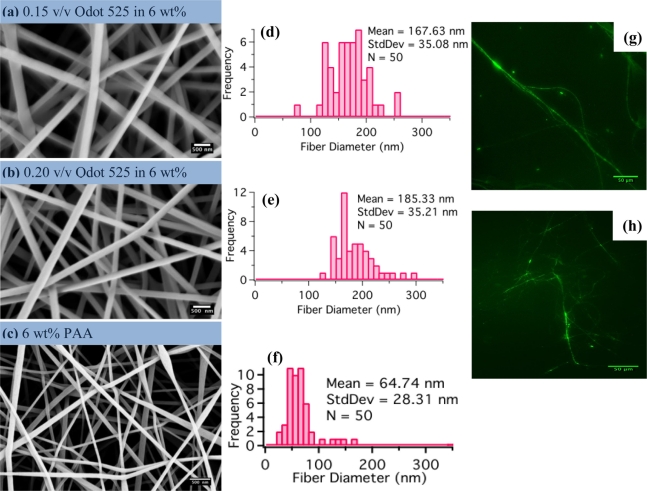
Composite fiber characterization: The SEM micrographs display smooth uniform diameter fibers for the composite fibers **(a,b)** and pristine fibers **(c)**. The mean fiber diameters for both composite populations **(d,e)** are within one standard deviation. The pristine fibers **(f)** are nanoscale but have spindle like defects. The composite fibers **(g,h)** are fluorescent as shown in the false colored fluorescence micrographs. (SEM scale bar = 500 nm and fluorescence micrograph scale bar = 50 μm).

Fibrous mats of composite SNP-PAA fibers were electrospun from aqueous solutions of PAA with Qdot 525s incorporated in the mixture. Mats were collected on both aluminum foil and waxed paper and spun on the same day under the same ambient conditions. The mats were refrigerated and stored in the dark and remained optically active for more than six months. Periodic fluorescence measurements on sections of the mats throughout the six months indicate the order of magnitude of the fluorescence remains the same, but due to variations in mat density and measurement technique it is difficult to determine if there was any appreciable degradation in the PL intensity. Fiber diameters and morphology were characterized with the SEM and shown in Figure 2(a,d). The processing conditions for all the mats as follows: 9 KV applied to the nozzle, the collector was 12 cm from the nozzle, and the spinning solutions was pumped at 0.9 mL/h.

**Figure 3.**
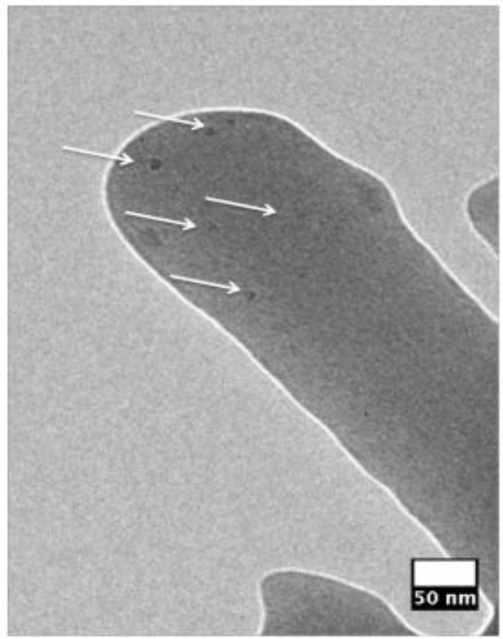
TEM micrograph of a composite fiber. Arrows are pointing to single and small clusters of SNPs in the fiber.

**Figure 4.**
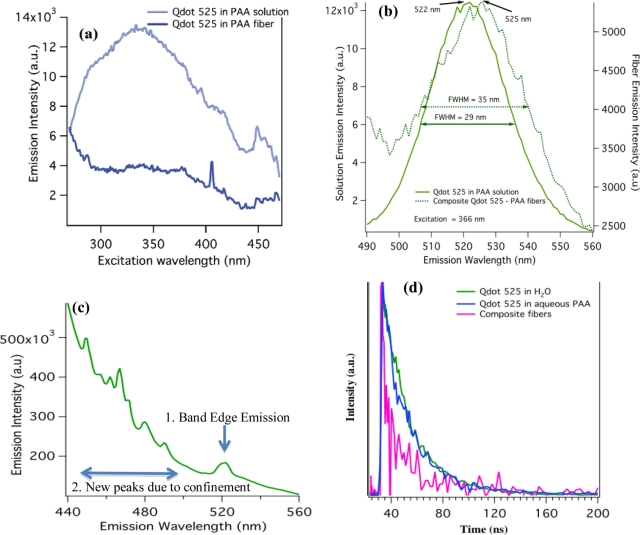
**(a)** Absorption (Photoluminescence Excitation); and **(b)** Band edge PL spectra (λ_ex_ = 366 nm) of 0.20% v/v Qdot 525 in aqueous PAA solution and composite fibers; **(c)** PL spectra (λ_ex_ = 405 nm) of composite fibers with new peaks due to confinement; **(d)** Fluorescence decay curves of 0.20% v/v Qdot 525 in aqueous PAA. The extracted excited lifetimes are as follows: 20 ns ± 2 ns, 19 ns ± 4 ns, and 14 ns ± 2 ns for the Qdot 525 in H_2_O, Qdot 525 in aqueous PAA and the composite fibers respectively.

In general, the SNP-PAA composite fibers are almost three times larger than pristine PAA fibers, shown in Figure 2(g), but have more uniform diameters. The TEM image in Figure 3 of a composite fiber reveals the SNPs are well dispersed in single dots and small clusters throughout the polymer matrix. Steady state fluorometry of the composite fibers shows the band edge emission PL peak is slightly red shifted (λ_sol_ = 522 nm to λ_fib_ = 525 nm) and the full-width half-max (FWHM) is wider for the fibers than the qdot 525-PAA aqueous solution [Figure 4(a,b)]. This change in the PL lineshape is expected if the SNPs are confined within the fiber [30]. Additionally, there are new higher energy peaks in the PL of the fiber mats that have been also shown by Demir *et al.* when quantum dots are confined in fibers that are smaller than the emission wavelength [27] (Figure 4(c). The excited lifetimes of the SNPs in water and the aqueous PAA-SNP solution are the same, 20 ns ± 2 ns and 19 ns ± 4 ns respectively but are reduced to 14 ns ± 2 ns in the fiber mat suggesting there is an a new relaxation pathway introduced by the fiber geometry (Figure 4(d). The optical microscopy, electron microcopy and fluorescence spectroscopy confirm that the SNPs are in the fibers and that the SNPs were still optically active after the electrospinning process.

### 3.2. Humidity and pH Sensing

**Figure 5.**
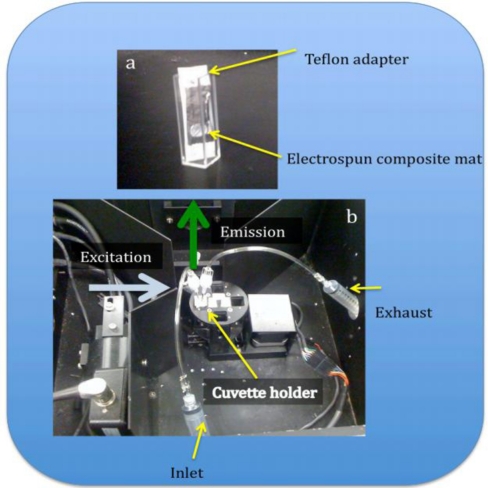
Photograph of the flourometer configured for sensing experiments. The cuvette **(a)** was modified with a Teflon adapter to secure the fibrous composite mat. For the humidity experiments, a 10 mL syringe was used to introduce the humid **(b)** air at the inlet and the second syringe was used to withdraw the air through the exhaust.

A steady state fluorometer was used for optical sensing of the PAA composite platform. The detection path was set at a 90° angle to the excitation path to improve the signal to noise ratio and a standard 10 mm path length fluorescence quartz cuvette was used for all the experiments. A custom Teflon film sample adapter was used to hold the composite mats at a 45° angle to the excitation [Figure 5(a)]. It was very important that the mats were secured in the same position during each pH series run, because the relative intensities changed if the mat moved, introducing error into the measurement.

PAA nanofibers have been demonstrated to swell mildly when immersed in alkaline solutions and shrink in acidic solutions [31]. Since the SNPs have shown sensitivity to the diameter of the fiber, we hypothesized that a dimensional change due to the environment would manifest in a change in the PL spectra of the fibers principally because the PAA fibers were not crosslinked, so we expected there would be a large change in diameter. However, in the absence of crosslinking, the PAA fibers were still soluble in water, limiting the choice of acids and bases. The fibers were insoluble in toluene (pH 7) providing a media for exposing the composite to range of pHs. Formic acid (pH 2), acetic acid (pH 4), triethylamine (TEA, pH 8) and pyridine (pH 8) were chosen for their solubility in toluene. The Qdots 525 were reported by the manufacturer to be stable in solutions from pH 2 to pH 9. The sequence of exposures in a series run are as follows: PL Spectra was taken of the dry mat, 2 mL of toluene was introduced to the cuvette containing the mat and another spectra was taken, 10 μL of acid or base was introduced and carefully stirred then PL spectra was taken, the toluene solution was pipetted out and the composite was rinsed with toluene and dried. The mat was never removed from the cuvette throughout the run limiting erroneous intensity fluctuations but the mat shifted when the TEA was removed resulting in a 38% decrease in the measured intensity. The sequence was repeated until the mat was exposed to the entire range of pHs. Each spectrum was recorded three times. Mats composed of 6 wt% PAA with 0.20% v/v Qdot 525 were used because they remained intact when peeled away from the wax paper.

**Figure 6.**
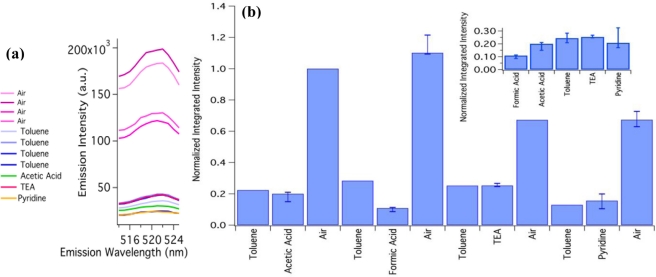
The normalized integrated intensity of the PL peaks to assess the response of the composite mat to changes in pH. Each blue bar represent the median area under the curve and the upper error bar represents the largest area and the lower error bar represents the lowest area in of the three spectra taken. The bars labeled Air represent the spectra taken after the mat was left to dry. Inset **(a)** displays the PL spectra curves. Inset **(b)** display the normalized integrated intensities in order of increasing pH. The toluene and pyridine spectra in this graph are adjusted by 38% to account for the overall decrease in intensity possibly caused by movement of the mat during the experiment.

To analyze the spectra, the area under the band edge emission peak [shown in Figure 6 inset (a), 514 nm to 526 nm] was calculated for each exposure then normalized to the integrated area of the first dry mat spectra (air). The results are shown in Figure 6. The biggest decrease in intensity came from submerging the mat in toluene, and remarkably the change in intensity was recoverable as seen in Figure 6, the second air spectra is the same intensity as the initial air spectra.

To determine if there was a significant difference in the intensities when the mat was exposed to the acids and bases we used a universal T-test with a 0.005 confidence interval and our hypothesis H_0_ is that μ_1_ = μ_2_. The results of the T-test are tabulated in Table 1.

There was a detectable difference in the intensity when the pH was changed from 2 to 8 and from 2 to 4, as well as from wet mats to dry. Otherwise the means of the populations were statistically the same within 95% confidence, suggesting the fluorescence is somewhat insensitive to small dimensional changes.

However, the effect on the hydration state of the composite was more pronounced. To explore this effect we configured the fluorometer as shown in Figure 5(b) and monitored only one wavelength (λ = 523) for 120 s while injecting and withdrawing moist air to and from the cuvette. Previous work by Meng *et al.* demonstrated that composite fibers of polystyrene and trioctylphosphine oxide (TOPO) terminated CdSe/ZnS increased fluorescence in the presence of water [32]. The authors suggested that the increase in fluorescence was due to the passivation of surface trap states by the water molecules. Our preliminary work saw an increase in fluorescence when the composite mats were released from a hydrophobic environment to a moisture rich environment. The ambient relative humidity was 61% on the day the pH tests were performed. We hypothesized that the fluorescence of our composite mats would also be sensitive to changes in relative humidity.

**Figure 7.**
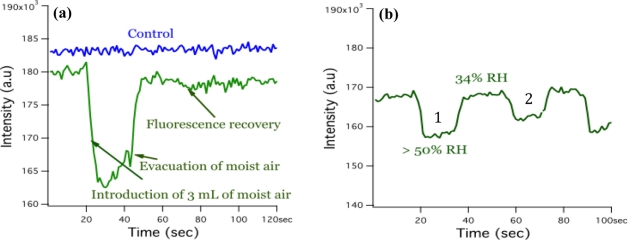
Plot of the magnitude change of the intensity of the band edge emission at λ = 523 when humid air is injected into the cuvette. **(a)** The plot on the right **(b)** illustrates the repeatability and volume sensitivity of the sensor’s response. In plot b, curve 1 represents two mL of moist air and curve 2 represents one mL of moist air.

The results of the humidity are shown in Figure 7. Surprisingly, the composite mat displayed a recoverable inverse relationship to humidity. As we increased the water content in the cuvette the fluorescence decreased as shown in Figure 7(a). Moreover the decrease in fluorescence was proportional to the amount of humid air introduced as illustrated in Figure 7(b) and was repeatable. The response time of the device on average was 5 s with a resolution of one reading per second. It is anticipated that single fiber response time would be much faster [32].

### 3.3. Sensing Mechanism

In analyzing the results from both the pH sensing experiment and humidity sensing experiment it is clear that the behavior of the polyelectrolyte in different environments is coupled to the exciton dynamics in the quantum dot. The pH sensing experiment hinted that the absorption of water by the composite fibers and the charge state of the polyelectrolyte influences the rate of radiative recombination in the quantum dots. The carboxyl group along the polyacrylic acid in the dehydrated state is protonated neutralizing the negative charge associated with the oxygen. When hydrated the carboxyl group deprotonates it becomes a charged anion. The SNPs are also functionalized with carboxylic acids to make them soluble in water and their charge state also depends on their hydration state.

**Figure 8.**
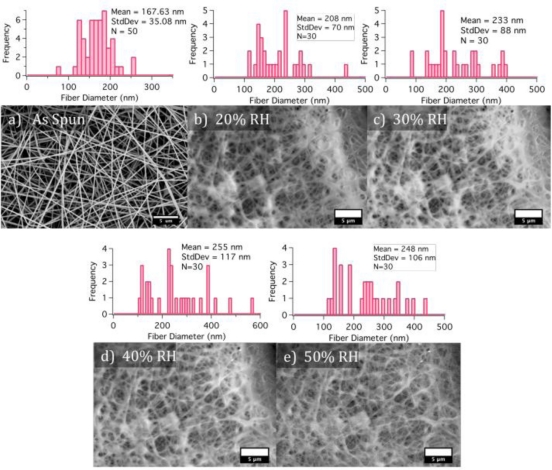
SEM micrograph **(a)** of the composite fibers imaged right after they were electrospun. Images **(b–e)** are ESEM micrographs of the composite fibers experiencing different ambient moisture contents.

**Figure 9.**
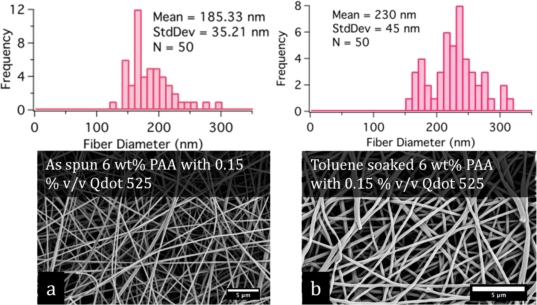
SEM micrographs of composite mats **(a)** as spun and **(b)** after soaking in toluene.

The pK_a_ of PAA is 4, meaning that at pH 4 less than half of the carboxylic acid groups are deprotonated. The measured pH of PAA and Qdot 525 in aqueous solution is 3, so in the presence of water, the polymer chain and Qdot 525s are experiencing some negative charge. To assess the hydration state of the composite fibers, ESEM was used to both image the composite fibers and control the relative humidity in the chamber. The composite fibers were imaged at 20, 30, 40 and 50% relative humidity (RH) (Figure 8), all conditions relevant to the experimental conditions, and the mean fiber diameters increased 24%, 39%, 53% and 48% respectively. The standard deviation of the fiber diameters for the 40% and 50% RH indicated a large spread so it is probable there is no difference between these two mats. Interestingly, a composite mat exposed to ambient 57% RH then soaked in toluene was imaged and the fibers were swollen 24% indicating there is still residual solvent in the fibers even after experiencing a vacuum (Figure 9).

In the pH sensing experiment, the fluorescence decreased when the mat was submerged in hydrophobic toluene but the fluorescence recovered somewhat as the pH was adjusted from 2 to 8 as shown in Figure 6. Adding a base increased the charge on the polymer by ionizing the carboxyl groups and the fluorescence recovered as seen in the formic acid- TEA comparison. To determine if there is residual water in the composite mats even after being soaked in toluene FT-IR spectra was taken on composite mats under the following conditions: (1) Composite mat dehydrated in a lyophilizer (Flexi-dry MP freeze-dryer, FTS Systems); (2) Composite mat exposed to ambient conditions; (3) Dehydrated composite mat soaked in toluene; and (4) Composite mat exposed to ambient conditions then soaked in toluene as was done in the sensing experiments; and (5) pristine PAA fibers (not shown).

**Figure 10.**
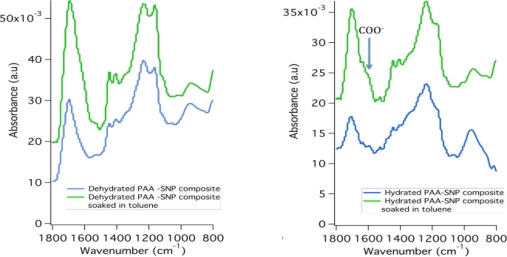
FT-IR spectra of the composite mats. In the spectra on the right, the presence of the shoulder indicated the carboxylic acids are deprotonated.

The FT-IR spectra (Figure 10) revealed the characteristic C═O peak at 1,700 cm^−1^ in all spectra but only the mats exposed to ambient conditions have the 1,543 cm^−1^ and 1,414 cm^−1^ peaks assigned to the antisymmetric COO^−^ and symmetric COO^−^ stretching, respectively. This verifies that even the mats soaked in toluene have water present and that the some of the carboxyl groups are deprotonated. In addition the 927 cm^−1^ peak assigned to the −OH out of plane bending associated with hydrogen bonding in PAA is very well defined in the fiber mats and is particularly pronounced in the hydrated PAA-SNP composite soaked in toluene suggesting the hydrophobic environment is sequestering the water and affecting the hydrogen bonding.

There is water present in both the dry composite mats and the mats saturated with toluene. We propose that the water in the presence of toluene is sequestered in pockets surrounded by hydrophilic carboxyl groups in the fibers [33]. These pockets also have a higher density of SNPs due to the hydrophilic functionalization on the surface on the SNPs. It is through the indirect interactions between the sequestered water, the polyelectrolyte and the SNPs in a hydrophobic environment that lead to unexpected fluorescence behavior in response to changing pH conditions.

If we consider the environment in these pockets when the PAA is electrostatically neutral, when the pH is adjusted to 2, the water molecules in the vicinity of the acidic hydrogen on both the SNPs and the PAA matrix form hydrogen bonds. Moreover, the water acts as an electron pair donor upon hydration of the carboxyl groups [34]. These liberated electrons are free to diffuse to the semiconductor surface forming a layer of negative charge sufficient to pull apart the photo excited electron hole pair causing a significant reduction of the radiative decay rate [35]. As the pH is increased, the carboxyl groups become ionized and the labile counter-ions have an increasing probability of becoming an electron acceptor reducing the charge around the SNP. This is manifest as a recovery of fluorescence as seen in the PL spectra when acetic acid is introduced. Further ionization of the carboxyl groups and the introduction of amines, which are also electron acceptors, leads to additional fluorescence recovery as seen when the fibers were exposed to TEA and pyridine.

The response of the composite in the humidity experiments is also attributed to the unique behavior of the water in the presence of the carboxylic acid groups. The FT-IR results indicated that the mats exposed to ambient conditions do have some deprotonated carboxyl groups but, as in the case of the acetic acid exposure, there are still a significant number of associated groups that will undergo hydrogen bonding with the water. As the water content in the air is increased, the PAA rapidly absorbs the water molecules increasing the hydration but not the dissociation of the carboxyl groups. As previously discussed this leads to an increase in free electrons in the matrix and a reduction in PL of the SNPs. When the water supply is decreased, the local environment reaches equilibrium and fluorescence is recovered.

## 4. Conclusions

This work has shown that electrospinning is a suitable process for making ultra-fine polyelectrolyte—SNP composite fibers. The SNPs retain their fluorescent properties and are embedded in the fibers. We have explored the charge state of the polyelectrolyte in the fibers and the unique electrostatic properties that arise from the ionizable groups on both the surface of the SNPs and the PAA. We have demonstrated the efficacy of using polyelectrolyte-SNP composites fibers sensing platform. Additionally, these composites provide an excellent venue for investigating the local environment long the polyelectrolyte chain and the interactions with the SNPs. Future work will be to model the proposed sensing mechanism.

## Figures and Tables

**Table 1.** Results of the T-test for H_0_ is μ_1_ = μ_2_.

**Test Pair**	**μ_1_**	**σ_1_**	**n_1_**	**μ_2_**	**σ_2_**	**n_2_**	**p**	**Accept**
Air-Acetic Acid	1.136	0.068	3	0.22	0.0264	3	0.0005	no
Air-Formic Acid	1.136	0.068	3	0.103	0.014	3	0.009	no
Air-TEA	1.136	0.068	3	0.255	1.010	3	0.0016	no
Air-Pyridine	1.136	0.068	3	0.22	0.026	3	.000078	no
Formic-Acetic Acid	0.103	0.014	3	0.22	0.264	3	0.006	no
TEA-Acetic Acid	0.255	0.01	3	0.22	0.264	3	0.133	yes
Pyridine-Acetic Acid	0.153	0.047	3	0.22	0.026	3	0.188	yes
Formic Acid-TEA	1.103	0.014	3	0.255	1.010	3	0.0002	no
Formic Acid-Pyridine	0.103	0.014	3	0.22	0.026	3	0.199	yes
TEA-Pyridine	0.255	0.010	3	0.153	0.047	3	0.058	yes
